# Cerebral Hyperperfusion After Hypoxic Brain Injury Secondary to Aspiration

**DOI:** 10.7759/cureus.91141

**Published:** 2025-08-27

**Authors:** Lisett Castellanos, Valentina Roa Forster, Mohammed Khatib, Mohammed S Uddin, Gabriela Perez

**Affiliations:** 1 Medicine, Florida International University, Herbert Wertheim College of Medicine, Miami, USA; 2 Neurology, Palmetto General Hospital, Miami, USA

**Keywords:** aspiration, cerebral blood flow, ct perfusion, focal neurological deficits, hyperperfusion, hypoxic brain injury, mean transit time

## Abstract

Hypoxic-ischemic brain injury, caused by inadequate oxygenation of the brain, may present as focal neurological deficits and can be associated with postischemic hyperperfusion of the affected tissue. An 84-year-old female patient was transferred to the hospital from a rehabilitation center with shortness of breath. One week prior, the patient aspirated food and was admitted to another facility, where this became complicated by altered mental status, catatonia, and left-sided weakness. Neuroimaging was negative, and she was discharged to a rehabilitation center. Upon arrival at the center, she developed shortness of breath that prompted her to come to our facility. On examination, she exhibited left-sided focal neurological deficits, stupor, and disorientation. Non-contrast brain CT revealed no acute abnormalities. However, CT perfusion imaging revealed increased cerebral blood flow and decreased mean transit time in the right hemisphere compared to the left. These imaging findings, in conjunction with her clinical presentation, are consistent with postischemic hyperperfusion secondary to hypoxic brain injury.

## Introduction

Hypoxic-ischemic brain injury (HIBI) occurs when the brain experiences a deficit in oxygen delivery, leading to neuronal injury and potentially cell death. The brain’s high metabolic demand makes it particularly vulnerable to hypoxia, even for brief periods [1,2]. The pathophysiology of HIBI involves a cascade of biochemical events that follow a hypoxic-ischemic insult. Lack of oxygenation forces cells to rely on anaerobic metabolism, resulting in reduced production of adenosine triphosphate (ATP) and a buildup of lactic acid. Without sufficient ATP to power energy-dependent pumps, the natural membrane gradient becomes disrupted, leading to the intracellular accumulation of sodium and calcium, as well as the loss of potassium. This imbalance results in excitotoxicity and the generation of free radicals. Altogether, this series of events yields necrosis and apoptosis of cells [3]. Even if blood flow is restored, there is a risk of reperfusion injury, which involves an influx of reactive oxygen species (ROS) and immune cells into the vulnerable brain tissue, potentially causing further damage [4]. On the other hand, reoxygenation of brain tissue can result in recovery and the return to neurologic baseline. This involves mechanisms such as neuroplasticity, the reorganization and remodeling of existing neurons, and neurogenesis, the generation of new neurons [5].

The clinical manifestations of HIBI vary widely depending on which region of the brain is affected and the severity of the insult. Presentations can range from asymptomatic to severe motor deficits to coma or even death [6]. Similarly, prognosis may also vary, from full recovery to long-term disability. Despite the use of physical examination and neuroimaging, accurately predicting the degree of recovery can be very difficult [7]. According to the American Heart Association (AHA), neurological prognostication in comatose survivors of HIBI, particularly following cardiac arrest, should not occur earlier than 72 hours after the return of spontaneous circulation [8]. Prognostic uncertainty may persist for days, weeks, or even months, as delayed recovery is possible. In intensive care settings where patients receive targeted temperature management (TTM) and sedation, the AHA recommends extending observation for at least one week after the completion of TTM or discontinuation of sedation to ensure accurate prognostication and account for delayed recovery [8]. Overall, prognostication in HIBI remains challenging, requiring both cautious interpretation and ongoing clinical observation. Integrating the patient’s history and clinical findings with these timelines is key to guiding timely diagnostic tests and management, which may greatly influence outcomes.

Common causes of HIBI include cardiac arrest, severe hypotension, and airway obstruction, such as aspiration. Aspiration occurs when a foreign object, often food, liquid, or even gastric contents, is inhaled and mechanically obstructs the respiratory tract [9]. This series of events leads to impaired gas exchange and, consequently, hypoxia, which can precipitate a brain injury [10]. Individuals at higher risk of aspiration include those with dysphagia, decreased levels of consciousness, impaired gag reflex, or neuromuscular weakness (e.g., myasthenia gravis). Older adults are particularly vulnerable due to age-related changes in swallowing coordination and reflexes [9].

Postischemic hyperperfusion, also called “luxury perfusion,” is a compensatory mechanism that can occur after HIBI, characterized by increased blood flow to an injured brain region [11]. This phenomenon can be detected using various neuroimaging modalities. On CT perfusion (CTP), it may appear as increased cerebral blood volume (CBV) and cerebral blood flow (CBF) on the injured side, contralateral to the presenting neurological deficits. Without careful correlation to clinical findings, postischemic hyperperfusion on imaging may be misinterpreted as ipsilateral hypoperfusion [12].

## Case presentation

An 84-year-old female patient with a medical history of diabetes mellitus, hypertension, hyperlipidemia, and extensive cardiovascular disease, including coronary artery disease with coronary artery bypass grafting, congestive heart failure, complete atrioventricular block with pacemaker placement, and severe aortic stenosis with aortic valve replacement, presented to the hospital with shortness of breath. One week earlier, she had choked on a piece of steak and aspirated. She was subsequently admitted to another hospital, where she was intubated and treated with antibiotics. Her hospitalization was complicated by altered mental status, catatonia, and acute left-sided weakness. According to the family, neuroimaging performed at the outside hospital was reported as negative for acute stroke; however, the imaging modality (CT or MRI) could not be confirmed. The patient was then discharged to a rehabilitation center on levetiracetam for seizure prevention. Upon arrival at the rehabilitation center, the patient developed shortness of breath. Her family reported that she had not received her regular dose of furosemide or levetiracetam. Emergency medical services (EMS) were called, and the patient was transferred to our facility. On arrival, she was stuporous, disoriented, and intermittently responsive. Furosemide and levetiracetam were administered. Immediately upon admission, the patient was taken for emergent neuroimaging.

While awaiting imaging results, a detailed neurological examination was performed: mental status (awake, alert, and oriented x3); cranial nerves (mild left-sided hemineglect); motor (left arm drift and hemiparesis); sensory (grossly intact, left leg monoplegia); reflexes (left-sided hyporeflexia with positive Babinski); coordination (unable to assess due to weakness). Levetiracetam was continued for seizure prophylaxis, although no clinical seizures were observed during her hospitalization.

Initial labs showed microcytic anemia (hemoglobin 8.1 g/dL, mean corpuscular volume 76 fL), hypoxemia with respiratory alkalosis (pH 7.51, partial pressure of carbon dioxide 32 mmHg, partial pressure of arterial oxygen 52 mmHg), and markedly elevated N-terminal pro-B-type natriuretic peptide (NT-proBNP, 12,900 pg/mL) with negative troponin, consistent with decompensated heart failure causing hypoxic respiratory failure. Additional findings included mild azotemia (blood urea nitrogen 24 mg/dL) and subclinical hypothyroidism (thyroid-stimulating hormone 5.2 µIU/mL, normal free thyroxine). Renal and hepatic function were preserved, and there was no laboratory evidence of infection, electrolyte imbalance, or metabolic disturbance, making these less likely contributors to her neurologic presentation.

**Figure 1:**
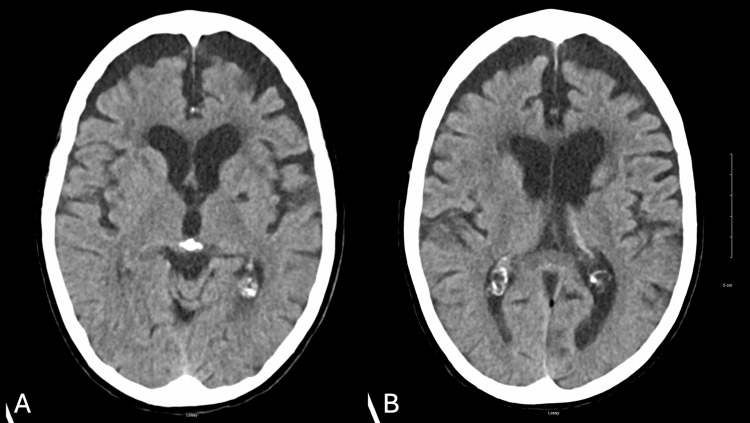
Non-contrast brain CT, axial view. CT taken during admission. No acute abnormalities noted. (A) Old lacunar infarct in left lentiform nucleus, moderate leukoecephalomalacia. (B) Asymmetric ventricles, left larger than right, moderate leukoencephalomalacia.

Non-contrast brain CT showed an old lacunar infarct in the left lentiform nucleus, asymmetric ventricles with left predominance, and moderate ischemic leukoencephalomalacia (Figures 1A, 1B).

**Figure 2:**
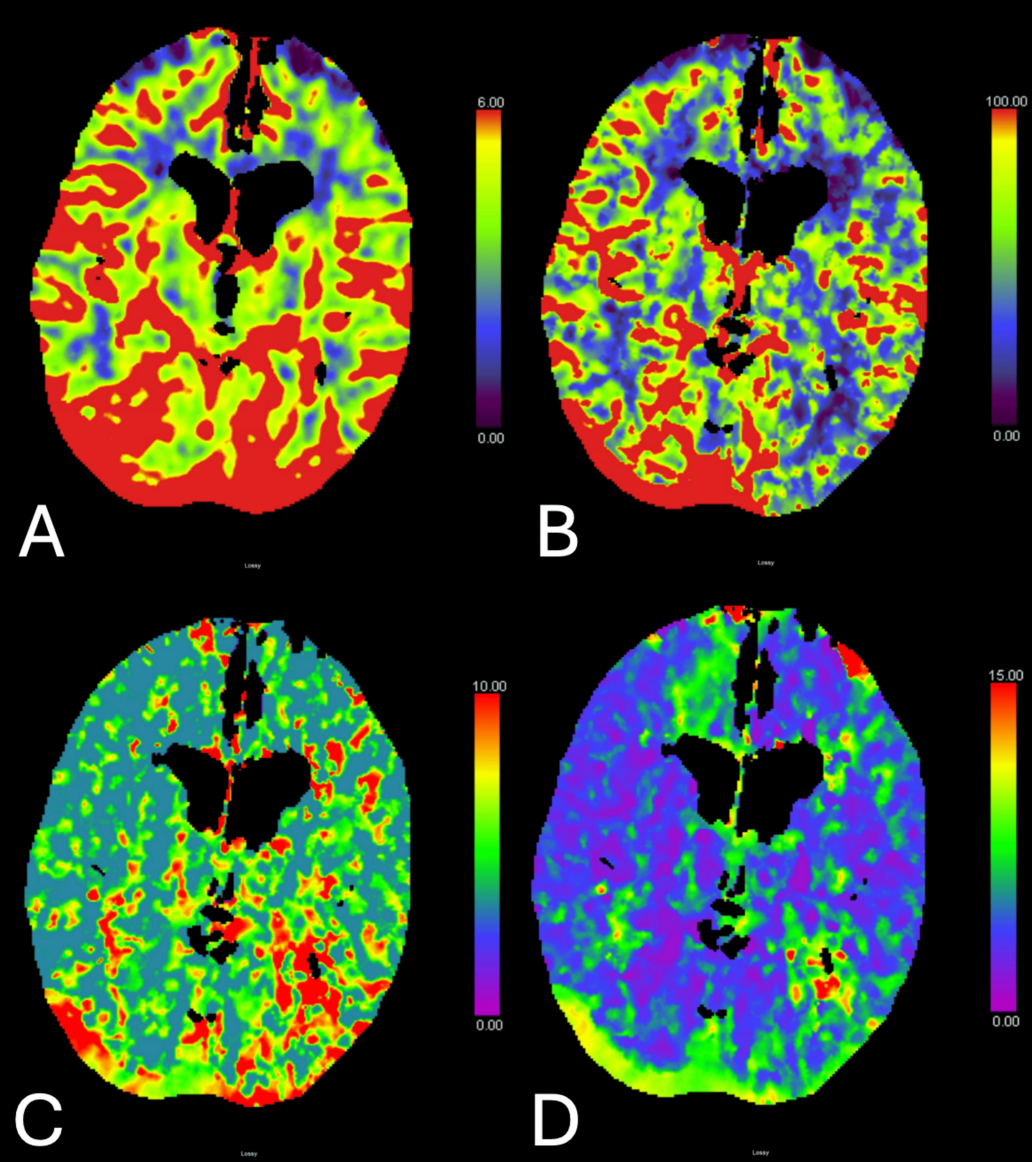
Brain CT perfusion (CTP) with contrast, axial view. CTP taken during admission. (A) Cerebral blood volume: increased on the right parietal lobe, appearing as red areas indicating higher blood volume. (B) Cerebral blood flow: increased on the right parietal lobe, shown as red areas corresponding to higher blood flow. (C) Mean transit time: decreased on the right parietal lobe, with blue areas representing shorter perfusion times. (D) Time to drain: decreased on the right parietal lobe, shown as blue areas indicating faster venous outflow.

No acute abnormalities were observed that could explain the patient’s current symptoms. CTP revealed increased CBF and decreased mean transit time (MTT) in the right cerebral hemisphere compared to the left (Figures 2A-2D). These findings were consistent with postischemic hyperperfusion. A brain MRI was not obtained due to concern that the patient’s pacemaker was MRI-incompatible. EEG revealed excessive bilateral and diffuse slowing of cortical rhythms, indicative of encephalopathy, without focal abnormalities or epileptogenic discharges. Analyzing the available results, we suspected that the patient was experiencing residual paralysis from hypoxia due to aspiration.

During hospitalization, the patient was managed with close neurological monitoring, seizure, fall, and aspiration precautions, and standard supportive measures to prevent secondary brain injury, including maintaining adequate perfusion and oxygenation. Over the course of her stay, she gradually regained strength in her left side and received standard inpatient rehabilitation with physical and occupational therapy to support functional recovery. She also received appropriate management for heart failure and respiratory failure, including oxygen therapy and diuretics. A follow-up non-contrast brain CT prior to discharge showed no acute changes. She was discharged with recommendations for outpatient follow-up with neurology, physical therapy, and her primary care provider.

## Discussion

This case illustrates the complexity of diagnosing HIBI, particularly when standard imaging modalities fail to reveal acute changes. The patient’s aspiration event likely led to hypoxia, resulting in right hemispheric brain injury, manifesting as left-sided neurological deficits. CTP provided valuable insight, revealing increased CBF and decreased MTT in the right hemisphere. These imaging findings are most consistent with the diagnosis of postischemic hyperperfusion, rather than acute stroke. If the patient were suffering an acute stroke, the alternative interpretation of CTP showing decreased CBF on the left hemisphere would not explain the patient’s left-sided weakness. Therefore, the most likely explanation of CTP findings is postischemic hyperperfusion. Although a brain MRI could have provided additional diagnostic certainty, it was not pursued due to concern that the patient’s pacemaker was MRI-incompatible. The combination of her clinical presentation and CT perfusion findings was considered sufficient to support the diagnosis. This phenomenon occurs as a compensatory mechanism to provide increased perfusion and, consequently, enhanced oxygenation to the injured brain region.

Laboratory findings supported acute decompensated heart failure as the cause of hypoxic respiratory failure, while excluding infection, electrolyte disturbance, or other metabolic derangements as contributors to her neurological deficits. Seizure activity was also considered but deemed less likely given that the patient was awake, alert, and steadily improving without seizure-like behaviors such as jerking movements, bowel or bladder incontinence, or tongue biting. EEG showed diffuse bilateral slowing without epileptiform discharges, consistent with encephalopathy rather than seizure, likely reflecting generalized cerebral dysfunction from HIBI. This correlation reinforces the importance of integrating neurophysiologic data with imaging and clinical findings in complex cases.

Understanding the parameters of CTP imaging is essential for accurate interpretation. CBF refers to the volume of blood moving through a given amount of brain tissue per unit time, typically measured in milliliters per 100 grams of tissue per minute (mL/100g/min). CBF reflects the rate of perfusion. CBV indicates the total volume of blood within a given region of brain tissue at a given moment, represented as mL/100g. This is indicative of the brain tissue’s vascular capacity. MTT is the average amount of time it takes blood to pass through a specific brain region, calculated by dividing CBV by CBF, and represented in seconds (sec). Time to peak (TTP) measures the time lapse between contrast injection to the point of maximum concentration in the tissue. TTP helps detect perfusion delay. These parameters collectively represent cerebral hemodynamics and aid in differentiating between ischemia, infarction, and postischemic hyperperfusion [13].

In acute infarction, both CBF and CBV are typically decreased, while MTT and TTP are prolonged. In an ischemic penumbra, or brain tissue that is potentially salvageable, CBF may be decreased while CBV is increased due to compensatory cerebral vasodilation. MTT and TTP remain prolonged in this scenario. Postischemic hyperperfusion, on the other hand, is characterized by increased CBF and CBV with normal or shortened MTT and TTP. This may be a beneficial compensatory mechanism, but it can also carry risk if excessive, such as reperfusion injury [11]. This occurs due to the sudden influx of blood with ROS and inflammatory cells that incite a series of biochemical events leading to further neuronal damage [14]. Hyperperfusion, particularly late onset, can even be a sign of impending hemorrhagic transformation [11]. Therefore, careful monitoring and clinical correlation are dire. Moreover, it will drive the kind of medical management that will be needed. This patient’s clinical improvement reflects a case of hyperperfusion that was beneficial, rather than harmful. Increased perfusion provided the necessary oxygen and nutrients to the sensitive brain tissue, allowing it to recover.

Although there is literature on postischemic hyperperfusion following cerebral ischemia due to stroke or cardiac arrest, limited information exists on this phenomenon in the setting of hypoxia secondary to aspiration [11,15]. Recognizing that hypoxic events can mimic cerebrovascular accidents is essential, especially in elderly patients who are at higher risk of aspiration. This case also adds to the literature by describing a case in which delayed hyperperfusion was identified and associated with clinical improvement. From a neurological standpoint, management was largely supportive, aimed at preventing secondary complications and promoting recovery. Outpatient follow-up with neurology and rehabilitation services was arranged to ensure ongoing recovery and monitoring. By recognizing postischemic hyperperfusion in this context, clinicians can avoid misdiagnosis of acute stroke, tailor monitoring strategies, and ensure appropriate neurological management.

## Conclusions

HIBI is a complex condition due to its diverse etiologies, broad and sometimes nonspecific clinical manifestations, and evolving radiographic findings. This case highlights the role of postischemic hyperperfusion in a patient with focal deficits and a recent history of hypoxia as a compensatory mechanism, demonstrated by CTP imaging. Recognizing this phenomenon helped differentiate it from an acute stroke, allowing for more accurate diagnosis and appropriate management. Recognition of postischemic hyperperfusion in patients with hypoxic events presenting with focal deficits may benefit future clinical care by informing diagnostic decisions, guiding monitoring and supportive measures, and ensuring appropriate neurological and rehabilitative follow-up.
